# A quantitative analysis of 3D printed face shields and masks during COVID-19

**DOI:** 10.35241/emeraldopenres.13815.1

**Published:** 2020-06-30

**Authors:** James I. Novak, Jennifer Loy

**Affiliations:** 1 School of Engineering, Faculty of Science, Engineering and Built Environment, Deakin University, Geelong, VIC, 3216, Australia

**Keywords:** Additive Manufacturing, Coronavirus, Fused Filament Fabrication, Maker Movement, Medical Product, Open Source, Pandemic, Personal Protective Equipment (PPE), Product design

## Abstract

In response to shortages in personal protective equipment (PPE) during the COVID-19 pandemic, makers, community groups and manufacturers around the world utilised 3D printing to fabricate items, including face shields and face masks for healthcare workers and the broader community. In reaction to both local and global needs, numerous designs emerged and were shared online. In this paper, 37 face shields and 31 face masks suitable for fused filament fabrication were analysed from a fabrication perspective, documenting factors such as filament use, time to print and geometric qualities. 3D print times for similar designs varied by several hours, meaning some designs could be produced in higher volumes. Overall, the results show that face shields were approximately twice as fast to 3D print compared to face masks and used approximately half as much filament. Additionally, a face shield typically required 1.5 parts to be 3D printed, whereas face masks required 5 3D printed parts. However, by quantifying the print times, filament use, 3D printing costs, part dimensions, number of parts and total volume of each design, the wide variations within each product category could be tracked and evaluated. This data and objective analysis will help makers, manufacturers, regulatory bodies and researchers consolidate the 3D printing response to COVID-19 and optimise the ongoing strategy to combat supply chain shortages now and in future healthcare crises.

## Introduction

The personal protective equipment (PPE) supply crisis caused by the COVID-19 pandemic ( Livingston *et al.*, 2020; Ranney *et al.*, 2020) has provided the 3D printing (aka. Additive manufacturing) community with a unique opportunity to utilise distributed networks of 3D printers to respond to local needs ( Larrañeta *et al.*, 2020; Tino *et al.*, 2020). Manufacturers with 3D printing capabilities, as well as individual makers operating from their homes, or community groups working from makerspaces, began responding to local shortages even before the World Health Organisation (WHO) declared COVID-19 a pandemic on 11 ^th^ March, 2020 ( Novak & Loy, 2020a). In the face of the emergency, the conventional pathways for product development and regulatory approval for use in healthcare settings were contracted or overwhelmed as the community response outpaced the ability for medical product regulators, like the Food and Drug Administration (FDA) in the United States or Therapeutic Goods Administration (TGA) in Australia, to respond.

Several months into the pandemic, interim guidelines have been refined ( Food and Drug Administration, 2020; Therapeutic Goods Administration, 2020); however, data shows that millions of 3D printed PPE products have been produced globally, extending beyond the capacity for regulators to manage. For example, IC3D Budmen self-reported that 3,026,172 of their face shields had been 3D printed at the time of writing ( Budmen, 2020), while 3D printer manufacturer Photocentric has been awarded a contract from The National Health Service (NHS) in the United Kingdom to 3D print over 7.6 million face shields in the coming months ( Hanaphy, 2020). Shared through online platforms where people collectively download, iterate, connect and learn how to assist their community ( Novak & Bardini, 2019; Özkil, 2017), the widespread 3D printing movement response to COVID-19 was unprecedented. As traditional manufacturing and supply chains stabilise through the middle of 2020, researchers must now provide governments, regulatory bodies and the broader 3D printing community with new insights that will guide proactive, long-term strategies to combat the long-term threat of COVID-19 and future health crises ( Gates, 2020).

Early COVID-19 research found that 60% of 3D printing projects responding to COVID-19 were for PPE ( Novak & Loy, 2020a), of which the top two products were face shields (62%) and face masks (20%). Representing 82% of all PPE products, this study focused on these two dominant categories of PPE to generate broad insights, as well as comparisons between two products that perform similar functions worn over the face. The principle aim was to quantify the qualities of face shield and face mask designs, in particular the print times, filament use, 3D printing costs, part dimensions, number of parts and total volume of each design. This builds upon a small study by Wesemann *et al.* (2020) of four COVID-19 face shields, which documented 3D printing data alongside qualitative feedback from ten clinicians. However, the low number of face shields analysed did not provide a broad understanding of the trends associated with this PPE category, and the designs documented have been superseded by updated versions.

As a case study on the role of iterative and open source development in 3D printing in the COVID-19 context, fused filament fabrication (FFF), also known as fused deposition modelling (FDM), was selected as the 3D printing technology to compare designs, building on emerging research in this area ( Larrañeta *et al.*, 2020; Tino *et al.*, 2020; Wesemann *et al.*, 2020). The focus was on the manufacturability of the designs, rather than a critical design engineering analysis of their relative effectiveness in use. The study provides a comparison between different designs, and tracks emerging trends in both their design development and adaption for manufacture. The results of this research will provide an objective review for those engaged in making, commissioning or using face shields or face masks that are in part, or predominantly, 3D printed.

While the circumstances of this situation are tragic, they highlight the fragility of globalised supply chains and the importance of establishing distributed manufacturing capabilities. The 3D printing industry and maker communities have argued that this technology and its collective capacity provide that capability ( Anderson, 2012; Gershenfeld & Cutcher-Gershenfeld, 2017; Lipson & Kurman, 2013). They have also driven open source development as a means to improve the design of products, particularly for the shared benefit of society. The COVID-19 pandemic has provided a unique opportunity to test many of these claims, and this paper provides a starting point for understanding the outputs of this approach and evaluate its viability.

## Methods

In order to conduct this analysis, STL files for face shields and face masks were downloaded from online sources. Designs were selected from two authorities: Firstly, face shields and face masks documented in Novak & Loy’s (2020a) critical review as being suitable for FFF were downloaded. This included face shields from 21 unique individuals, groups or companies published online prior to 1 ^st^ April 2020, and 7 face masks. Where possible, multiple versions of files were accessed in order to gain information about how designs had matured over time. It is important to note that only respirator-style face masks covering the nose and mouth were included in this study, with full face masks such as modified snorkelling masks, or small 3D printed additions for conventional cloth masks, discounted from results due to the large variations in scale and complexity.

Secondly, the COVID-19 database on the National Institutes of Health (NIH) 3D Print Exchange was used to download FFF designs that had been approved for either “clinical use” or “community use” by 30 ^th^ May 2020. The NIH 3D Print Exchange is a biomedical 3D file sharing community that established a specific COVID-19 evaluation platform in collaboration with the Veterans Healthcare Administration to qualify designs, acting as an authority for 3D printing that provided some level of validation to the plethora of open source designs being shared online ( NIH 3D Print Exchange, 2020). While many more designs had been uploaded to the NIH 3D Print Exchange than those approved, as well as numerous other 3D file sharing communities, it was important to focus on designs which had gained traction in the 3D printing community through validation and testing, similar to the projects documented by Novak & Loy (2020a) which had spent several months online being validated and peer reviewed through use.

Each design was then sliced in Cura (v.4.6.1) in preparation for 3D printing. This allowed vital information to be collected about the estimated print time and amount of filament used, without the necessity to physically produce such a large number of products. The settings used for slicing each design are listed in Table 1, with the settings reflecting a typical <$1000 desktop FFF machine without hardware modifications, upgrades or other features that may allow faster printing speeds or larger nozzle diameters. This reflects the conservative capabilities of many 3D printers being used by makers to produce face shields or face masks during the COVID-19 pandemic. While support material was not required for most parts, Table 1 also shows the settings used in several cases where production would not be possible without supports, with these designs marked in the *Underlying data* ( Novak & Loy, 2020b).

**Table 1.** Principle Cura print settings used for slicing.

Layer height	0.25mm
Line width	0.4mm
Wall thickness	1.2mm
Top/bottom thickness	0.75mm
Infill density	20%
Infill pattern	Zig Zag
Print temperature	235°C (PETG)
Build plate temperature	75°C
Top/bottom print speed	30mm/s
Print speed	50mm/s
Travel speed	100mm/s
Build plate adhesion	None
Supports*	None*
**If supports necessary**	
Placement	Touching build plate
Overhang angle	45°
Pattern	Zig Zag
Density	15%

Autodesk Meshmixer (v.3.4.35) was used to collect additional geometric data about each design, including the measurements of the largest part for each project in order to understand the size of the 3D printer required to produce it. The volume of each individual part was also calculated, resulting in a total volume for all parts required to produce a single product. This collection of data allowed for quantitative analysis of the broad trends and features of both 3D printed face shields and face masks.

## Results

In total, 37 separate designs or versions of face shields were collected from 24 individuals, groups or companies, documented in full in *Underlying data* ( Novak & Loy, 2020b). This was three more sources on top of those originally reported by Novak & Loy (2020a), having been uploaded to the NIH 3D Print Exchange from the 1 ^st^ April 2020. 31 separate designs or versions of face masks were also collected from 14 sources, which included 7 additional individuals, groups or companies from Novak & Loy’s (2020a) study.

**Figure 1.**
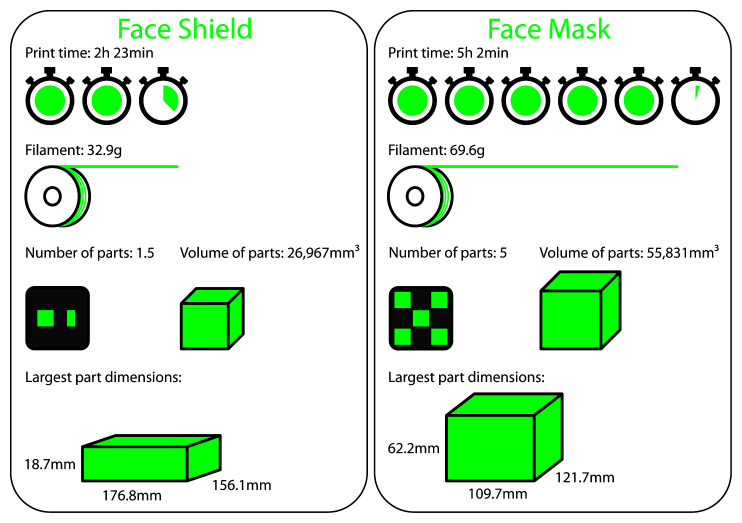
Average data for face shields and face masks.

Average data for the 37 face shields and 31 face masks is shown in Figure 1, with a trend for face masks to take approximately twice as long to print compared to face shields, being made up of approximately twice as much volume, and requiring approximately twice as much filament. The median values for face shields were: print time (2h 15min), filament (31g), number of parts (1), volume of parts (25,775mm ^3^) and largest part dimensions of 185.8 x 145.6 x 20mm (XYZ). The median values for face masks were: print time (4h 29min), filament (60g), number of parts (3), volume of parts (47,089mm ^3^) and largest part dimensions of 109.0 x 125.3 x 58.8mm (XYZ).

**Figure 2.**
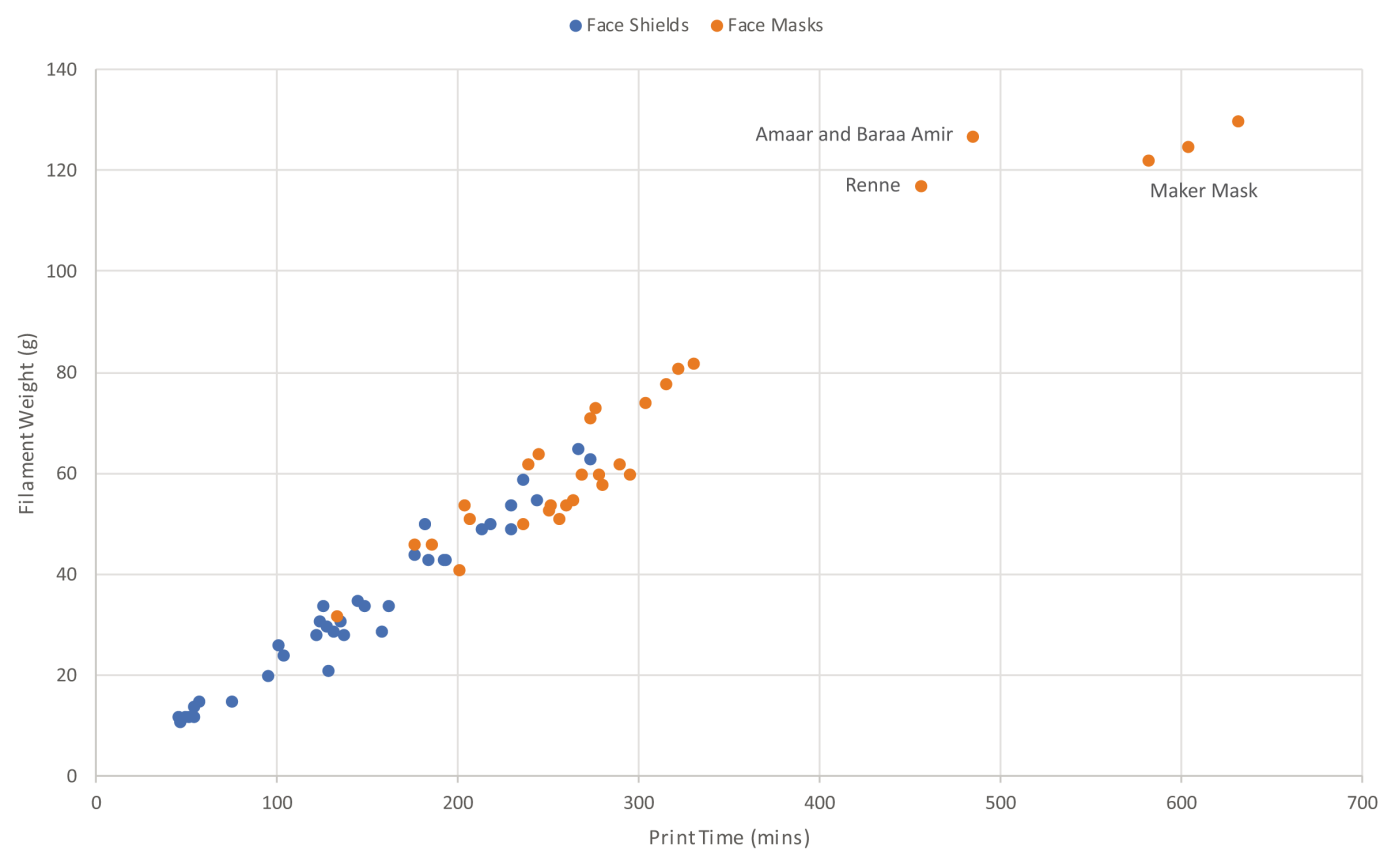
Print time versus filament weight for face shields and face masks.

**Figure 3.**
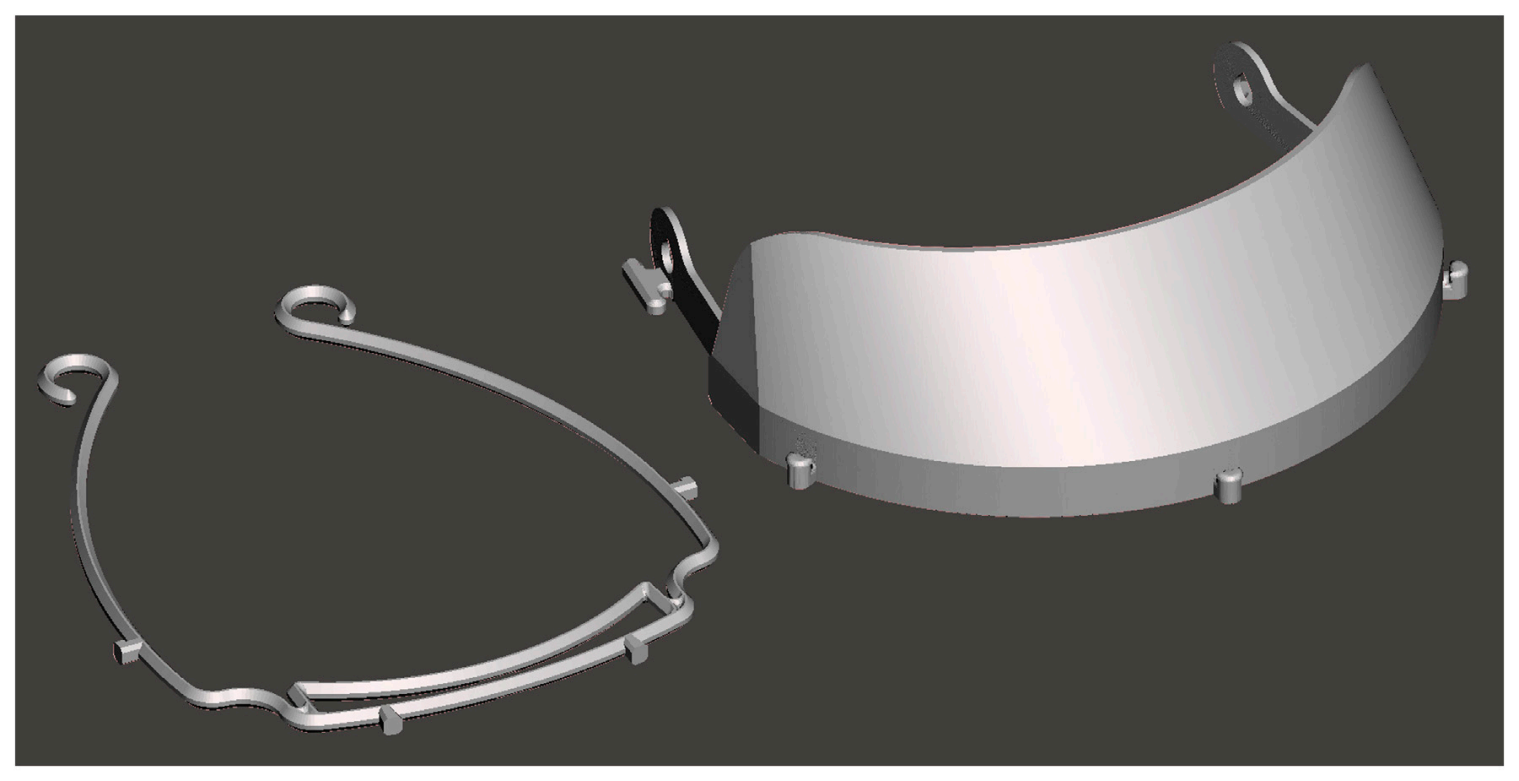
Version 1 face shield from MSD Robotics Lab (left) compared to the design from MITRE Corporation (right).

Figure 2 shows all 68 designs plotted against the print time and filament weight to produce them, with a clear trend for increased filament to be required the longer a design takes to produce. Increased filament use is directly related to increased cost. For face shields, the shortest print time was 46mins to produce a single part with 12g of material for the Version 1 face shield from MSD Robotics Lab. However, the current version 3 design at the time of writing recorded an increased print time of 54mins, using the same amount of material in slightly different proportions. The longest print time for a face shield was 4h 34min (274min) and required 63g of filament, also only a single part from MITRE Corporation. These two designs are shown in Figure 3.

**Figure 4.**
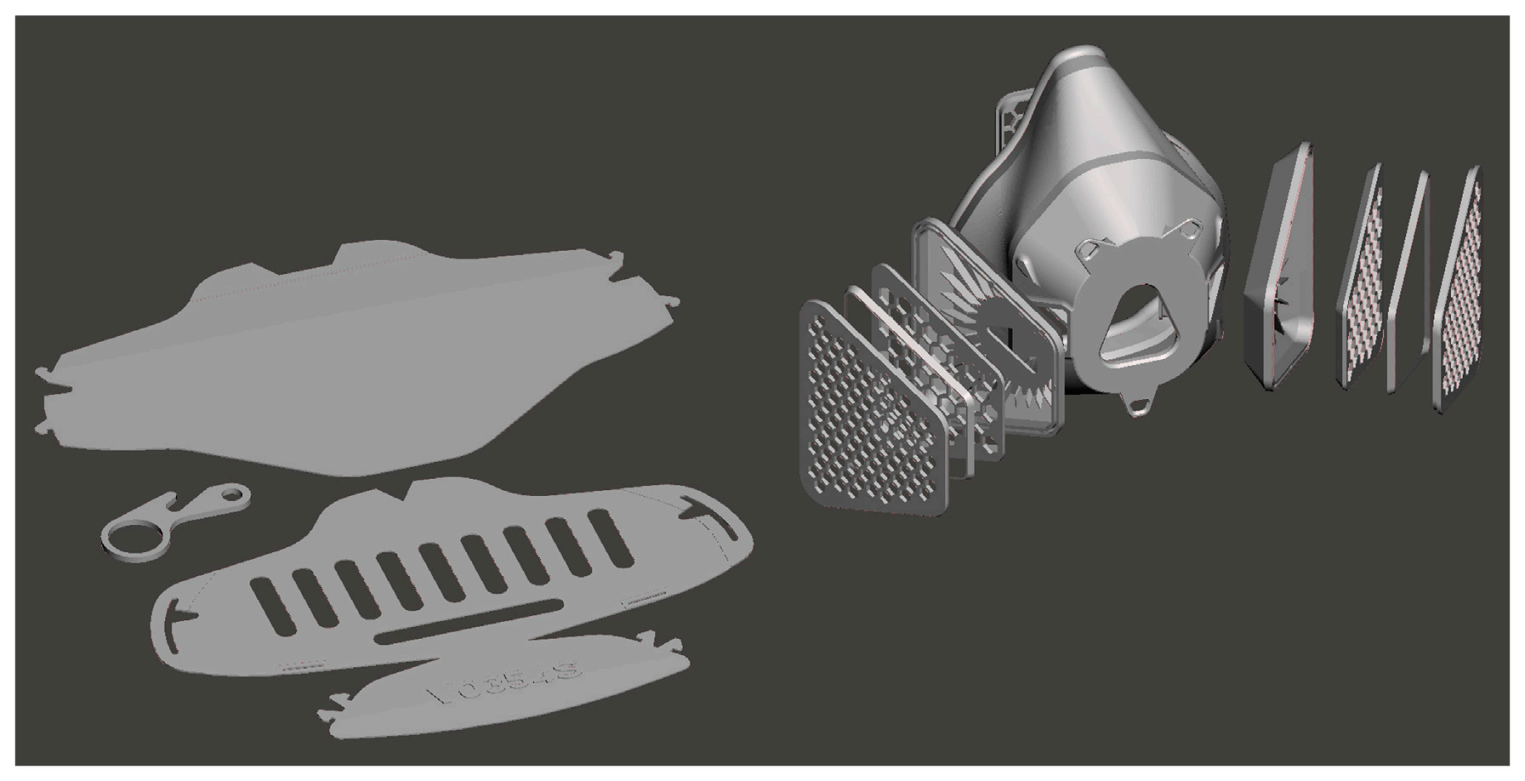
Version 0.354 face mask parts from Collective Shield (left) compared to the Respirator V2 from Maker Mask (right).

For face masks, the shortest print time was 2h 14min (134mins) requiring 32g of filament for a 3-part design from Collective Shield (v.0.354). This design is 3D printed in a flat form only 0.6mm thick and then folded into a 3D face mask, often referred to as a “2.5D print” ( Kandemir *et al.*, 2018; Novak *et al.*, 2019), as shown in Figure 4. Because of the flat nature of this design, it also reported the largest width dimension of 244.3mm, requiring a larger 3D printer build plate than any other face mask or face shield. In contrast, the longest print time for a face mask was 10h 32mins (632mins) with 130g of filament required to print 26 separate parts, forming a respirator style mask called Respirator V2 from Maker Mask. This design is also shown in Figure 4. The earlier Respirator V1 required 24 parts and took 10h 4mins (604mins) to 3D print, while a different style of mask from the Maker Mask organisation called Rapid V1 only took 4h 16mins (256mins) and 51g of filament to produce, consisting of 3 parts.

**Figure 5.**
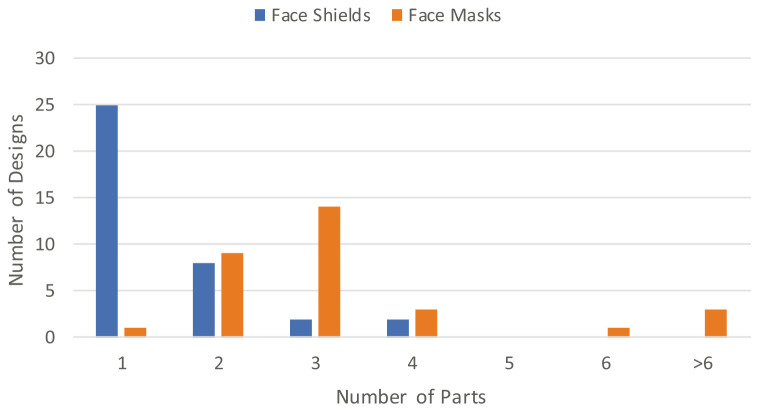
Number of 3D printed parts required to produce each face shield and face mask.

As visualised in Figure 1, the average number of 3D printed parts required to produce a single face shield was 1.5, while for face masks it was 5. However, as shown in Figure 5, 68% of face shields only required a single part to be 3D printed, with the maximum number of 3D printed parts found for any design being 4. Yet for face masks, only one design could be produced from a single 3D print, with 45% of face masks requiring 3 3D printed components. There was one face mask that required 6 components, before 3 designs from Maker Mask that required a large number of separate 3D printed parts mentioned previously, with the addition of a Children’s V1 mask that also required 26 components like the Respirator V2. These outliers are clearly visible at the extreme end of Figure 2, and are responsible for skewing averages calculated in this study.

**Figure 6.**
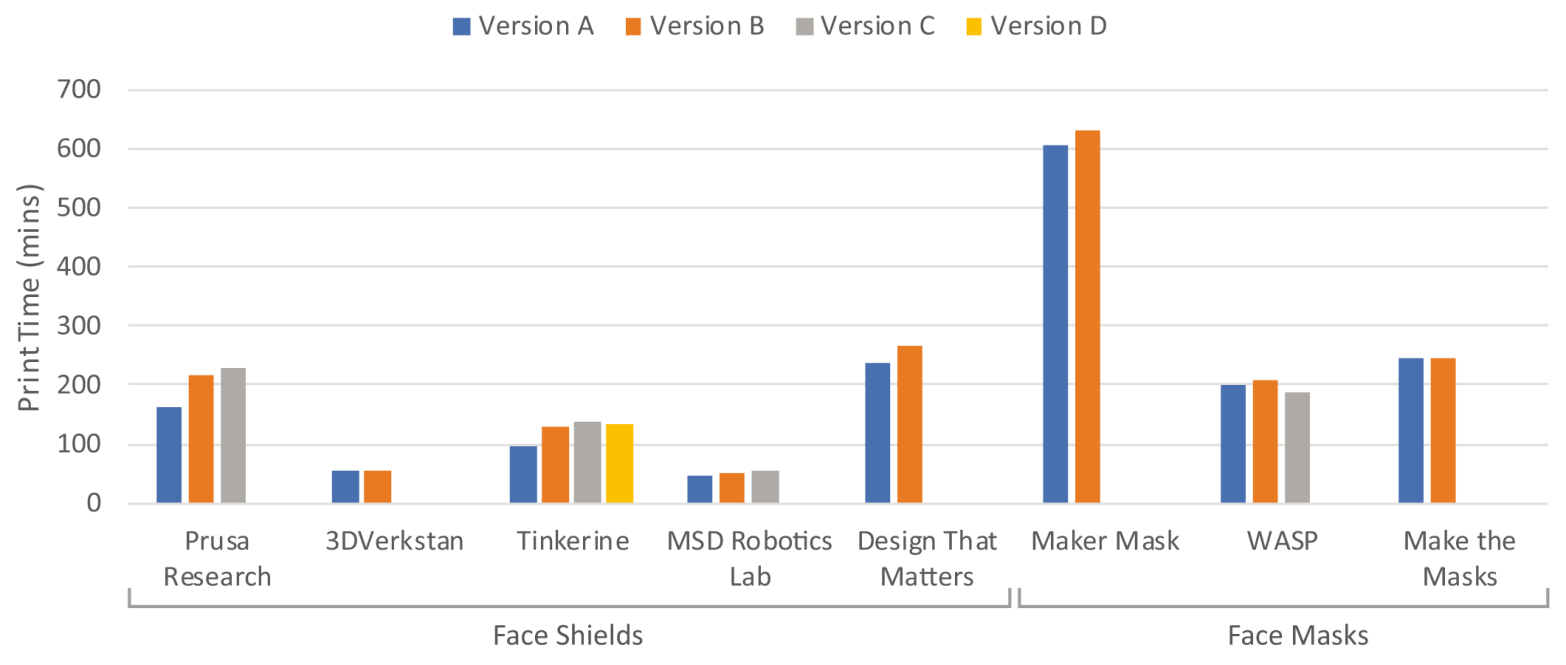
Comparison of print times for different versions of face shields and face masks.

As mentioned previously, several versions of face shields and face masks were analysed in this study, representing improvements made during the course of the COVID-19 pandemic based on feedback from the maker community and healthcare workers. Shown in Figure 6 is a comparison of the print times for versions of the same designs, with “Version A” representing the first online release still available for download at the time of writing, for example the RC1 face shield from Prusa Research. There was a common trend for print times to increase with design revisions, suggesting that parts gained additional features, or expanded in volume over time. Only the 3DVerkstan face shield and Montana V2 face mask from Make the Masks remained identical in print time and filament use between versions with only minor geometrical differences, while the latest face shield from Tinkerine and face mask from WASP experienced a reduced print time compared to the previous versions. It must be noted that other designs may have gone through numerous versions, however, were not available for download.

**Figure 7.**
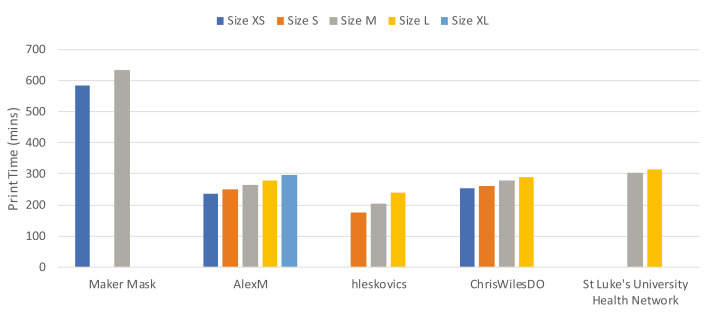
Comparison of print times for different sizes of face masks.

A trend that was unique to face masks was for a single design to be available in multiple sizes, as documented in Figure 7. Unlike face shields which were largely noted to be one-size-fits-all, with any adjustment made through the addition of elastic or the inherent flexibility of the design, face masks must conform to the face of a wearer in order to provide any protection from airborne particles. Therefore, the availability of various sizes was observed for 5 out of the 14 (36%) projects in this study, with the face mask by AlexM having the largest number of sizes including XS, S, M, L and XL. The increasing print times in Figure 7 directly corresponded to increased filament required to produce each mask, and therefore cost.

Assuming a price for PETG filament of $30/Kg, the cost of 3D printed components for face shields can be calculated to range from $0.33–1.95, while the range of face masks was $0.96–3.90. For one-off products these differences may not be critical to makers, yet when multiplied by hundreds of thousands or even millions, the potential investment by makers, organisations, charities and businesses may vary significantly based on the selection of one design over another, or one version of a design over another.

## Discussion

With research showing that 60% of 3D printing projects responding to COVID-19 during the first months of the pandemic were for PPE ( Novak & Loy, 2020a), with this category also one of the most discussed on social media ( Vordos *et al.*, 2020), this quantitative data provides a more detailed insight into the 3D print and geometric qualities that makers need to consider. Overall data from this study shows that face shields are significantly quicker to 3D print than face masks, requiring less material, less 3D printed parts, and therefore costing less to 3D print, which may be contributing factors to the popularity of face shields amongst makers compared to face masks.

As shown in Figure 2, a cluster of 8 face shields recorded print time less than one hour, with the V1 design from MSD Robotics Lab recording the shortest time of 46mins. However, as a superseded version, with V3 from MSD Robotics Lab requiring 54mins to print, the shortest print time of a current version face shield at the time of writing was the V14 design from FabLab Benfica, taking 47mins to print. Compared to the slowest face shield from MITRE Corporation, 5.8 of the FabLab Benfica design could be produced in the same time, while compared to the quickest face mask, 2.9 face shields could be printed in the same time. Within the face mask category, 4.7 of the Collective Shield design could be printed in the same time as a single Maker Mask Respirator V2. For makers looking to produce products in maximum quantity, this comparative data is particularly useful to understand.

The method of this study, which utilised consistent process parameters for each design, provides accurate comparative data between designs and versions of a design; however, it is important to note that many of the projects specify unique print settings in order to maximise print speeds or other qualities. For example, the 3DVerkstan face shield describes that it was optimised to be 3D printed with a ≥0.8mm nozzle and layer thickness >0.3mm ( 3DVerkstan, 2020) which would significantly reduce print time, reportedly to less than 20mins per part, compared to the 54mins using a smaller 0.4mm nozzle and settings in this study. Similarly, according to Prusa Research (2020), the RC3 face shield should be quicker to 3D print than the previous RC2 version, which was not found to be true with the settings in this study. This highlights the influence of design for additive manufacturing (DfAM), when a product has been designed for a specific machine or process ( Diegel *et al.*, 2019; Novak & O'Neill, 2019; Thompson *et al.*, 2016), as well as the importance of print settings to optimise production ( Chua & Leong, 2017). Yet with the global nature of the maker community, equipped with a broad variety of FFF 3D printers with different capabilities, it was important to provide objective and realistic data aligned with the capabilities that many makers may have, particularly those who may have less experience in modifying machines to print with large nozzles, or modifying slicing settings to get the most out of their machine for a specific design.

While the data collected in this study may not align with individual claims made online, it is validated by the smaller study of Wesemann *et al.* (2020) of 3D printed face shields, which used similarly standard print settings to produce several designs. They recorded print times for the Prusa Research RC1 and RC2 of 2h 30mins and 3h 17mins respectively, and filament use of 30g and 42g, compared with print times of 2h 42mins and 3h 38mins and filament use of 34g and 50g using the settings in this research. Wesemann *et al.* (2020) also recorded a print time for the V3 iteration of the IC3D Budmen design of 2h 6mins with 33g of filament, compared with the V4 iteration in this study of 2h 15min and 31g of filament. While similar, the data shows how different makers will experience different outcomes for the same design, making online reporting and interpretation challenging due to the plethora of printers, and different versions of printers, available globally. This is an ongoing challenge for distributed additive manufacturing systems with “the sheer variety in machines, materials and processes mak[ing] the development of a uniform standard for AM a challenging task” ( Gao *et al.*, 2015).

One of the methods to speed up production of PPE products not investigated in this research is stacking, particularly for face shields. Several designs are available pre-stacked for download, with claims that this increases throughput of machines. For example, the Prusa Research RC3 is available as a file containing 4 face shield headbands stacked on top of each other, which when printed on a well-calibrated Prusa 3D printer, reportedly reduces print times to 1h 20mins each ( Prusa Research, 2020). It must be noted, however, that the website warns users that their printer must be very well calibrated, and to expect their 3D printer to be louder than normal during operation. The smaller 3DVerkstan face shield ( 3DVerkstan, 2020) is provided in several stack sizes up to a stack of 52 pieces measuring 290mm tall, meaning that printers capable of printing this height can be left operating over night without any need for a maker to manually remove individual parts and restart the machine every hour or less. These files were not included in this study due to the complexity of comparing dissimilar prints with multiple parts stacked in different configurations, however, could form a future research study. In order to meet supply chain shortages, such innovative print methods are important to understand, particularly in maximising the small build plates of most desktop FFF 3D printers.

Additional to the print time, it is also important to acknowledge that this study did not consider the entire time required to produce a face shield or face mask, including fabrication of other components, assembly, postprocessing and sanitisation. This data could change the preference of makers looking for the fastest items to produce and deliver to healthcare workers, particularly in the face mask category where more parts are typically required compared to face shields, and more skill may be required to adapt and assemble filtration features or flexible materials to ensure accurate fitting between the mask and a user’s face. This study also did not consider the human aspect of these products, for example comfort or protective qualities, and future research may find that some of the fastest items to 3D print were not preferable for healthcare workers to wear for long durations. Data from Wesemann *et al.*, (2020) supports this, indicating through a survey of 10 clinicians that the Prusa Research RC1 face shield, while quicker to print than the RC2, was less comfortable and less fitting, despite offering the same level of protection. In responding to the healthcare crisis, balance must be found between production time and comfort, and there is no point 3D printing the design that is quickest if healthcare workers cannot comfortably and safely wear it for long periods of time. In the long-term, as 3D printing allows for design innovation, the development of more appropriate designs informed by expertise in the technology should be addressed ( Loy, 2015).

**Figure 8.**
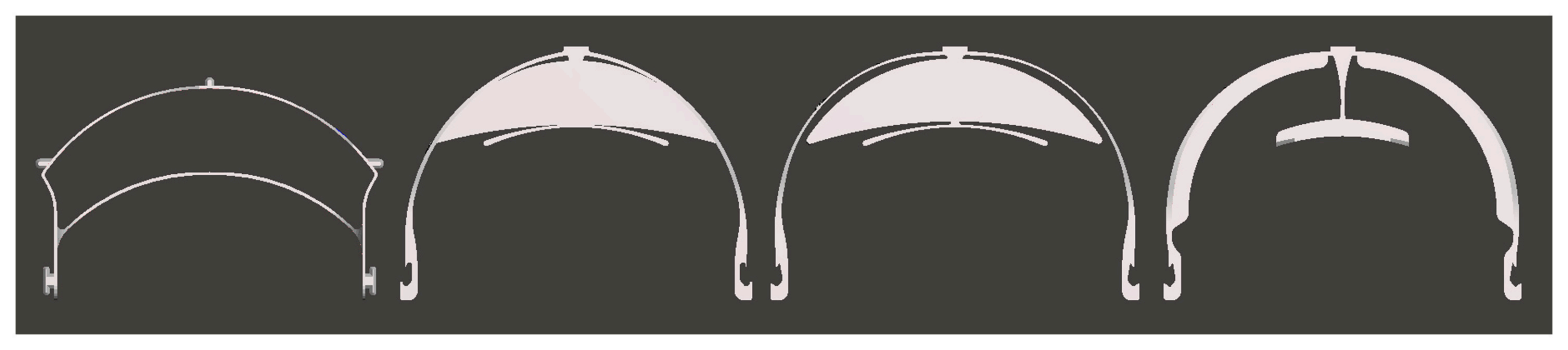
Tinkerine face shield versions from left to right: V1, V3, V4, V8.

In response to such feedback from clinicians, as well as the maker community, several designs showed changes to print times with each iteration, visualised in Figure 6. While some changes were minor, some designs changed quite substantially. For example, the Tinkerine versions in Figure 8 show a progression from initially being a completely 3D printed design from V1 to V4, before evolving by V8 to include space for foam to be added in the space between the frame and the wearer’s forehead to maximise comfort and create a better seal against airborne particles. At the time of writing the design had progressed to V9.4 which only utilised several small 3D printed clips, taking 17mins to print, with foam being used to form the main structure ( Suyu, 2020). This meant that face shields could be produced more rapidly than relying entirely on 3D printing for the frame. However, due to the design and manufacturing shift away from 3D printing, this version was not included in the results to ensure comparisons remained between similar designs using 3D printing in similar ways. Future studies must look at additional fabrication methods and may compare 3D printed designs with other manufacturing methods, particularly with a view to finding the quickest and safest design to produce en masse.

**Figure 9.**
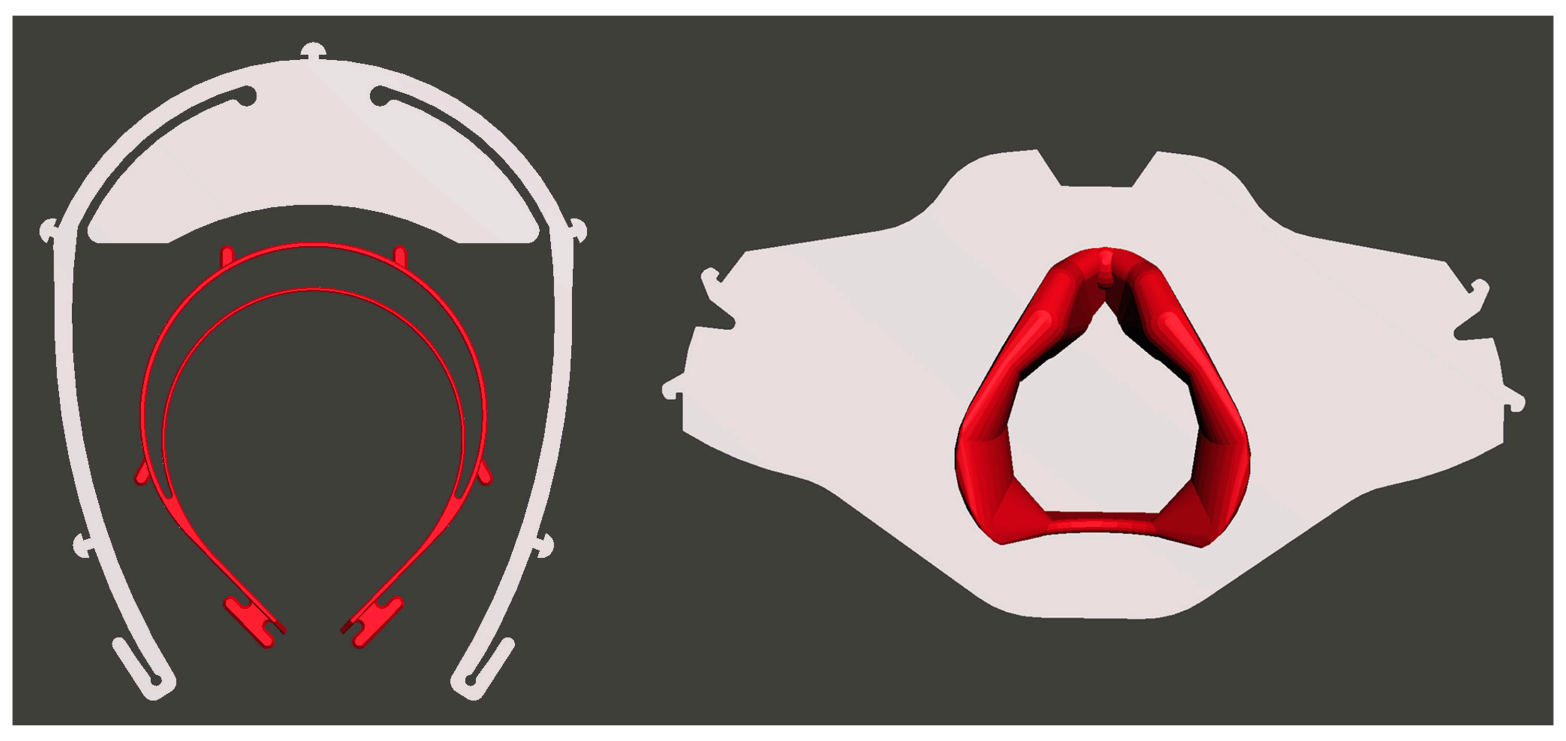
Comparison of small and large designs: on the left is a the Prusa Research RC1 (red) compared to the Aon3D R01 (grey), and on the right is the largest component of the a26 Helmet Compatible v5.2 (red) compared to the largest component of the Collective Shield V0.354 (grey).

A final factor that makers must consider is the geometry of the design; specifically, the overall size of parts compared to the build volume of their 3D printer ( Redwood *et al.*, 2017). While the data in the results section provides average and median values for the largest component of a design, the breadth of sizes is more visually represented in Figure 9. The Prusa Research RC1 was one of the smallest face shield designs, measuring 120 × 136 × 20mm, whereas the Aon3D R01 face shield was one of the largest measuring 182 × 219 × 3mm. For many desktop FFF machines, build plates are <200mm ( Redwood *et al.*, 2017), meaning that several designs, including the Aon3D face shield, cannot be produced. Therefore, makers may gravitate to smaller designs out of necessity, not just because of print times. Similarly, the largest component of the a26 Helmet Compatible v5.2 face mask was 84 × 85 × 33mm, fitting within even the smallest build volume, compared to a Collective Shield V0.354 part measuring 244 × 132 × 0.6mm. While fastest to produce, the Collective Shield design prohibits many makers due to the large size that does not fit common build volumes.

For the future of distributed manufacturing, based on 3D printing, this example of practice demonstrates the principle of collaborative, iterative design development. It also highlights the challenges of working across different brands and models of FFF machines when optimising a product for batch production. Whilst it is hoped that the need for PPE that drove the condensed development pathway of these products has eased, it may be that the experience provides the foundations for developing more responsive, agile manufacturing systems where new products can be collectively designed, tested, and adapted in real-time.

## Conclusion

As makers around the world responded to supply chain challenges associated with COVID-19, quantifying the differences between the increasing number of solutions, particularly PPE, which is relied upon by healthcare workers and the broader community for protection, is important. This study demonstrates the range of considerations for makers when selecting a design to 3D print, including their 3D printer hardware, the quantity of parts to be produced, the amount of filament required and the resulting cost. Some of these parameters can be controlled and modified through process parameters, however, many are directly attributed to the original designer’s intent and their ability to optimise a design for the fused filament fabrication process.

One of the overall findings from the 37 face shields and 31 face masks was that face shields take approximately half as long to 3D print as face masks and use approximately half as much filament, requiring on average 1.5 3D printed parts compared to 5 for face masks. This directly translates to face shields being cheaper to 3D print than face masks. Within a product category the variations are significant, with print times measured in hours between different designs that perform the same function, varying the amount of filament required, and therefore the cost by several dollars per unit. As the 3D printing community transitions from reacting to the COVID-19 pandemic to more consolidated and proactive measures, understanding these trends can help designers and regulators better optimise parts for distributed manufacturing in a global health crisis.

The example of 3D printing for the COVID-19 PPE crisis provides the justification of investigations into the use of distributed manufacturing, enabled by this technology. This paper contributes to building not only a body of knowledge specific to the localised manufacture of face shields and face masks, but also to the broader sustainability argument for a rethink of centralised manufacturing. The COVID-19 PPE supply experience demonstrates the potential to develop realistic alternatives, and the results of this study illustrate that new ways of building validated procedures are both needed, and possible.

## Data availability

### Underlying data

Figshare: Underlying Data: A Quantitative Analysis of 3D Printed Face Shields and Masks During COVID-19, https://doi.org/10.6084/m9.figshare.12496520.v1 ( Novak & Loy, 2020b).

This project contains the following underlying data:

- COVID-19 PPE Data - Face Masks.csv
- COVID-19 PPE Data - Face Shields.csv

Data are available under the terms of the Creative Commons Attribution 4.0 International license (CC-BY 4.0).
