# Supplementary material for: A quantitative analysis of 3D printed face shields and masks during COVID-19
Source: Emerald Open Res. 2020 Jun 30;2:42. doi: 10.35241/emeraldopenres.13815.1 (PMC7391928; doi:10.35241/emeraldopenres.13815.1)
Supplement: Peer review history

# Peer review: Reviewer response for version 1

**DOI:** 10.21956/emeraldopenres.14893.r26991

**Published:** 2020-07-29

**Authors:** Nick Vordos

**Affiliations:** 1 Department of Physics, International Hellenic University, Kavala, Greece

The article is a study of qualitative analysis of personal protective equipment created during the COVID- 19 pandemic. It is a well-written article that has studied 37 different designs of face shields and 31 face masks created with common (not-professional) 3D printers. Time, cost, filament weight and other parameters were studied.

 What is missing from the study:

 The authors did not refers the type of filament used. Also, a comparison of different filaments (ABS, PLA, ASA, PET, PP, etc) will be useful. Finally, it is important to state the reason for the exclusion from the study of the resin printing technique.

Is the work clearly and accurately presented and does it cite the current literature?

Yes

If applicable, is the statistical analysis and its interpretation appropriate?

Not applicable

Are all the source data underlying the results available to ensure full reproducibility?

No source data required

Is the study design appropriate and is the work technically sound?

Yes

Are the conclusions drawn adequately supported by the results?

Yes

Are sufficient details of methods and analysis provided to allow replication by others?

Yes

Reviewer Expertise:

Nanostructural Characterisation, Bioengineering,

I confirm that I have read this submission and believe that I have an appropriate level of expertise to confirm that it is of an acceptable scientific standard.

# Peer review: Reviewer response for version 1

**DOI:** 10.21956/emeraldopenres.14893.r26951

**Published:** 2020-07-29

**Authors:** Allan E. W. Rennie, H Kursat Celik

**Affiliations:** 1 Akdeniz University, Antalya, Turkey; 2 Engineering Department, Lancaster University, Lancaster, UK

This is a well-researched and documented review of a targeted selection of the plethora of PPE products (face shields and masks) that emerged mostly as a ‘call-to-arms’ from the maker community and/or the low-cost/desktop AM technology providers amidst the COVID-19 pandemic. The key parameters considered within the study include the fabrication time, material utilisation as both mass and volume, number of constituent components and the overall envelope for the product (dimensions in x, y, z) – it is appreciated that cost (of the stock material) could be subject to variations based on geographical location of the person/organisation making the PPE products. This is key to providing a good understanding of the practicalities of using additive manufacturing/3D printing for the fabrication of this type of product as it very often dispels the myth/hype that is prevalent regarding the suitability of the technology for such a task (in lieu of the more conventional, higher volume production techniques such as injection moulding, whilst manufacturing organisations gear up/commission tooling, etc and then the unit price and production time per item becomes more realistic).

 Although the authors make reference to the interim guidelines published by the FDA (USA) and TGA (in Australia), it may be useful to acknowledge (where possible) the adherence (or otherwise) of other guidelines/standards that were applicable to the PPE products that were evaluated, based on their country of origin – this could be tabulated for easy reference by the wider readership should they wish to seek out applicable guidelines in their location.

Is the work clearly and accurately presented and does it cite the current literature?

Yes

If applicable, is the statistical analysis and its interpretation appropriate?

Yes

Are all the source data underlying the results available to ensure full reproducibility?

Yes

Is the study design appropriate and is the work technically sound?

Yes

Are the conclusions drawn adequately supported by the results?

Yes

Are sufficient details of methods and analysis provided to allow replication by others?

Yes

Reviewer Expertise:

Additive manufacturing, engineering design.

We confirm that we have read this submission and believe that we have an appropriate level of expertise to confirm that it is of an acceptable scientific standard.
